# ZEB1 Modifies VE‐Cadherin Signaling in Lymphatic Endothelial Cells

**DOI:** 10.1111/micc.70076

**Published:** 2026-06-25

**Authors:** Nada S. Ahmed, Joseph L. Horder, Charles T. Cresswell, Poppy E. Harris, Amy P. Lynch, Zarah B. Tabrizi, Kathryn R. Green, James H. Hallwood, Anton C. Smith, Michael A. Portelli, Alexander J. Fezovich, Christos Spanos, David S. Gardner, Alan McIntyre, Sarah J. Storr, Daniel G. Booth, David O. Bates, Andrew V. Benest

**Affiliations:** ^1^ Endothelial Quiescence Group, Centre for Cancer Sciences, School of Medicine, Biodiscovery Institute University of Nottingham Nottingham UK; ^2^ Faculty of Pharmacy Ain Shams University Cairo Egypt; ^3^ Tumour Vascular Biology Laboratories, Centre for Cancer Sciences, School of Medicine, Biodiscovery Institute University of Nottingham Nottingham UK; ^4^ Centre for Respiratory Research, National Institute for Health Research Nottingham Biomedical Research Centre, School of Medicine, Biodiscovery Institute University of Nottingham Nottingham UK; ^5^ Hypoxia and Acidosis Group, Centre for Cancer Sciences, School of Medicine, Biodiscovery Institute University of Nottingham Nottingham UK; ^6^ Wellcome Discovery Research Platform for Hidden Cell Biology University of Edinburgh Edinburgh UK; ^7^ School of Veterinary Medicine and Science University of Nottingham Nottingham UK; ^8^ Nottingham Breast Cancer Research Centre, School of Medicine University of Nottingham, Biodiscovery Institute University Park UK; ^9^ Cell Division and Chromosome Structure Laboratory, Centre for Cancer Sciences, School of Medicine, Biodiscovery Institute University of Nottingham Nottingham UK

## Abstract

**Objective:**

Zinc finger E‐box‐binding homeobox 1 (ZEB1) is a transcription factor primarily known for its regulatory roles in epithelial‐to‐mesenchymal transition (EMT) and cell fate determination. Recent studies suggest that endothelial ZEB1 signaling promotes blood vessel growth and reduces junctional integrity, although the underlying mechanisms remain unclear. Notably, the role of ZEB1 in the lymphatic vasculature is unknown, and the regulation of lymphatic integrity by VE‐cadherin remains poorly defined.

**Methods:**

Here, using an integrated proteomic and transcriptomic approach, we identify ZEB1‐dependent signaling pathways associated with cell–cell junction reorganization in lymphatic endothelial cells (LECs).

**Results:**

Loss of ZEB1 reduced VE‐cadherin phosphorylation at pY731 and pY685 and was accompanied by decreased monolayer resistance and impedance, together with increased leukocyte transendothelial migration. ZEB1 knockdown also reduced YES tyrosine kinase expression and altered YAP1 expression and junctional localisation, changes that were associated with reduced VE‐cadherin phosphorylation. Silencing YAP1 in HDLECs similarly reduced VE‐cadherin phosphorylation and impaired barrier integrity, recapitulating aspects of the phenotype observed following ZEB1 knockdown.

**Conclusions:**

Collectively, these findings suggest that ZEB1 contributes to lymphatic endothelial barrier maintenance in association with altered YAP1 and YES signaling.

## 1 Introduction

The endothelium forms the inner lining of both blood and lymphatic vessels, resulting in an interface between the vessel contents (blood or lymph) and surrounding interstitial spaces. This position exposes endothelial cells (ECs) to a range of mechanical and biochemical stimuli, leading to the emergence of distinct functional specializations and dynamic EC phenotypes, including organ‐specific, arterial, venous, proliferative, and activated states [1]. The blood vasculature primarily delivers oxygen and nutrients to tissues, whereas the lymphatic vasculature serves as a drainage and immune surveillance system that clears excess fluid, protein, and immune cells [2]. Under physiological conditions, the endothelium remains largely quiescent, maintaining tight regulation of cell adhesion, proliferation, motility, and junctional integrity. Controlled modulation of quiescence, and therefore junctional condition, is essential for controlling the passage of cells, solutes, and fluid across the endothelium [3, 4, 5].

The lymphatic endothelium is a specialized endothelium [2], composed of lymphatic endothelial cells (LECs) that possess transcriptomic characteristics distinct form blood ECs, while retaining a clear EC identity [6]. Within lymphatic capillaries, LECs form discontinuous, button‐like junctions that facilitate the uptake of interstitial fluid and macromolecules. In contrast, collecting lymphatic vessels display continuous zipper‐like junctions that support efficient lymph transport [7]. Inter‐LEC junctions comprise tight junctions (TJ), adherens junctions (AJ), and connexin‐comprising gap junctions. Disruption of barrier integrity is associated with the loss of endothall quiescence, while precise regulation EC permeability is essential for maintain tissue homeostasis [8].

VE‐cadherin is widely regarded as the master regulator of endothelial junction integrity. VE‐cadherin signaling is modulated by phosphorylation at specific tyrosine residues within its cytoplasmic tail. In particular, phosphorylation of Y685 and Y731 has been shown to regulate vascular permeability and leukocyte extravasation. These phosphorylation events are controlled by tyrosine kinases, including YES [9], and counterbalanced by phosphatases such as VE‐PTP [10, 11] and SHP‐2 [10]. However, most studies of VE‐cadherin signaling have focused on the blood vasculature, and considerably less is known about how these regulatory pathways operate in lymphatic endothelial cells.

Zinc‐finger E‐box binding protein 1 (ZEB1; also known as δEF1, TCF8, Zfhep and Zfhx1a) is a transcription factor that has been extensively studied in cancer, where its canonical roles include promoting epithelial–mesenchymal transition (EMT) and tumor invasivenesss [12]. In endothelial cells, ZEB1 signaling contributes to endothelial‐to‐mesenchymal transition (EndoMT) [13], and mathematical modeling predicts that reduced ZEB1 activity promotes vessel destabilization during angiogenesis [14] suggesting that loss of endothelial ZEB1 may increase EC plasticity. Consistent with a role for ZEB1 in maintaining endothelial quiescence, increased vascularity has been reported in xenografted tumors grown in globally ZEB1‐heterozygous mice [15], alongside evidence of disrupted VE‐cadherin–mediated Wnt signaling during development [16] and hyperproliferation of corneal ECs [17]. However, other studies indicate context‐dependent roles for endothelial ZEB1. For example, endothelial ZEB1 is required for normal bone development [18], EC‐specific ZEB1 deletion promotes vessel normalization in tumor‐bearing mice [18], EC‐specific ZEB1 knockout promotes vessel normalization in tumor bearing mice [18] and models of corneal disease require ZEB1 for angiogenesis‐like remodeling [19, 20]. To date, there are no reports describing whether ZEB1 promotes or attenuates endothelial stability within the lymphatic vasculature, despite substantial evidence linking ZEB1 to the regulation of endothelial quiescence. Here, we used an integrated transcriptomic and proteomic approach to investigate the mechanisms of ZEB1 action in human dermal lymphatic endothelial cells (HDLECs), identifying a central role for ZEB1 in junctional remodeling.

## 2 Methods

### 2.1 Cell Culture and Maintenance

Pooled Primary Human Dermal Lymphatic Endothelial Cells (HDLECs, Promocell RRID:SCR_023579, Heidelberg, Germany) were cultured in endothelial cell growth medium MV2 (Promocell, Heidelberg, Germany) supplemented with MV2 supplements (Promocell, Heidelberg, Germany) in a humidified environment with 5% CO_2_ at 37°C. Cells were passaged at 80%–90% confluency and cells were reseeded or pelleted at an appropriate density for each experiment, and used between P1‐5. HDLECs are of juvenile foreskin (dermal) origin and are confirmed free of mycoplasma. Cell identity was verified by the supplier to be Pecam and Podoplanin positive and has not been reported misidentified.

### 2.2 Small Interfering RNA Mediated Transfection

RNAi using 20 nmol ON‐TARGETplus (SMARTpool Dharmacon) to ZEB1 (6935, L‐006564‐01‐0020, target sequences: CUGUAAGAGAGAAGCGGAA, CUGAAAUCCUCUCGAAUGA, GCGCAAUAACGUUACAAAU, GCAACAGGGAGAAUUAUUA). YAP1, L‐012200‐00‐0020, ON‐TARGETplus Human YAP1 (10413) siRNA—SMARTpool Dharmacon GCACCUAUCACUCUCGAGA, UGAGAACAAUGACGACCAA, GGUCAGAGAUACUUCUUAA, CCACCAAGCUAGAUAAAGA And siControl (ON‐TARGETplus non‐targeting pool) (D‐001810‐10‐20, Dharmacon UGGUUUACAUGUCGACUAA, UGGUUUACAUGUUGUGUGA, UGGUUUACAUGUUUUCUGA, UGGUUUACAUGUUUUCCUA). 1 × 10^6^ HDLECs per 60 mm dish and left to adhere overnight. Cells were transfected with 20 nM of siRNA human ZEB1 and YAP1 (denoted as ZEB1 KD and YAP1 KD respectively) and control cells with non‐targeted siControl (denoted as NS) using Lipofectamine RNAimax (Thermo Fisher Scientific) for 6 h. The transfection reagent complex was then aspirated and replaced with MV2. Cellular material was extracted after 48 (RNA) or 72 (protein) hours.

### 2.3 RNA Extraction and Sequencing

RNA extraction was performed using RNeasy Mini Kit (Cat No. 74104, QIAGEN). RNA concentration was quantified using Nanodrop, and all samples were deemed pure if 280/260 ratio > 1.75. RNA samples were diluted to 5 μg using RNAase‐free water. First strand cDNA was synthesized using random hexamer primer and M‐MuLV Reverse Transcriptase (RNase H‐). Second strand cDNA synthesis was subsequently performed using DNA Polymerase I and RNase H. Double‐stranded cDNA was purified using AMPure XP beads. Remaining overhangs of the purified double‐stranded cDNA were converted into blunt ends via exonuclease/polymerase. After adenylation of 3′ ends of DNA fragments, NEBNext Adaptor with hairpin loop structure was ligated for hybridisation. cDNA fragments of 150–200 bp in length were generated by PCR and purified using the AMPure XP system (Beckman Coulter, USA). Transcriptomes were read by Illumina HiSeq platforms by Novogene Inc. (Hong Kong, China) in a paired‐end manner to a depth of 20 M reads.

Read quality and adapter content were assessed with FASTQC (v0.11.9) [21]. Reads were trimmed of adapters and quality filtered using cutadapt (v4.4) [22] to a minimum quality 20 (−q 20) and a minimum read length of 1 in a paired‐end manner. Subsequent alignment of trimmed, paired‐end reads was performed using STAR (v2.7.9) to the reference genome GRCh38 (primary assembly, release 107, https://ftp.ensembl.org/pub/release‐107/fasta/homo_sapiens/dna/). Alignment files were output from STAR (v2.7.9) in sorted BAM format. Aligned reads were quantified using featureCounts (v2.0.0) [23]. Read normalization and identification of significantly (Benjamini‐Hochberg adjusted *p*‐value < 0.05) [24] differentially expressed genes (DEGs) was carried out with DESeq2 (v1.36.0) [25] under R (v4.2.0, https://www.R‐project.org/). DEGs were imported to WEB‐based GEne SeT AnaLysis Toolkit (WebGestalt, https://www.webgestalt.org/#) and Qiagen Ingenuity Pathway Analysis (IPA) [26] to identify the most significant enriched biological processes, cellular components and molecular functions in ZEB1 knockdown cells as per Pinel et al. [27].

### 2.4 Proteomics

Protein and peptide lists generated using the same software and the same parameters. Specifically, 35 μg of total protein from each sample were digested using the Filter Aided Sample Preparation (FASP) protocol as described by Wisniewski et al. [28] with minor modifications. In brief, each protein sample was added onto 30 kDa MWCO filter units (Vivacon, UK) along with 150 μL of denaturation buffer (8 M Urea in 50 mM ammonium bicarbonate [ABC] [Sigma Aldrich]) and centrifuged at 14000×*g* for 20 min, while another wash with 200 μL of denaturation buffer was performed under the same conditions. The protein samples were then reduced by the addition of 10 μL of 10 mM dithiothreitol (Sigma Aldrich, UK) in denaturation buffer for 30 min at 22°C, and alkylated by adding 100 μL of 55 mM iodoacetamide (Sigma Aldrich, UK) in denaturation buffer for 20 min at ambient temperature in the dark. Two washes with 100 μL of denaturation buffer and two with digestion buffer (50 mM ABC) were performed under the same conditions described above before the addition of trypsin (Pierce, UK). The protease:protein ratio was 1:50 and proteins were digested overnight at 37°C. Following digestion, samples were spun at 14000×*g* for 20 min and the flow‐through containing digested peptides was collected. Filters were then washed one more time with 100 μL of digestion buffer and the flow‐through was collected again. The eluates from the filter units were acidified using 20 μL of 10% Trifluoroacetic Acid (TFA) (Sigma Aldrich), and spun onto StageTips as previously described by Rappsilber [29]. Peptides were eluted in 40 μL of 80% acetonitrile in 0.1% TFA and concentrated down to 1 μL by vacuum centrifugation (Concentrator 5301, Eppendorf, UK). The peptide sample was then prepared for LC–MS/MS analysis by diluting it to 5 μL by 0.1% TFA.

LC–MS analyses was performed using an Orbitrap Exploris 480 Mass Spectrometer (Thermo Fisher Scientific, UK) coupled on‐line, to an Ultimate 3000 HPLC (Dionex, Thermo Fisher Scientific, UK). Peptides were separated using 50 cm (2 μm particle size) EASY‐Spray column (Thermo Scientific, UK), which was assembled on an EASY‐Spray source (Thermo Scientific, UK) and operated constantly at 50°C. Mobile phase A consisted of 0.1% formic acid in LC–MS grade water and mobile phase B consisted of 80% acetonitrile and 0.1% formic acid. Peptides were loaded onto the column at a flow rate of 0.3 μL min^−1^ and eluted at a flow rate of 0.25 μL min^−1^ according to the following gradient: 2%–40% mobile phase B in 150 min and then to 95% in 11 min. Mobile phase B was retained at 95% for 5 min and returned back to 2% after 1 min, until the end of the run (190 min).

Survey scans were recorded at 120000 resolution (scan range 350–1650 m/z) with an ion target of 5.0 × 10^6^, and injection time of 20 ms. MS2 Data Independent Acquisition (DIA) was performed in the orbitrap at 30000 resolution with a scan range of 200–2000 m/z, maximum injection time of 55 ms and AGC target of 3.0 × 10^6^ ions. We used HCD fragmentation [30] with stepped collision energy of 25.5, 27, and 30.

The DIA‐NN software platform (version 1.8.1) [31] was used to process the raw files and search was conducted against the *Homo sapiens* Uniprot database (released in May, 2019). Precursor ion generation was based on the chosen protein database (automatically generated spectral library) with deep‐learning based spectra, retention time and IMs prediction. The digestion mode was set to use trypsin, allowing for up to two missed cleavages. Carbamidomethylation of cysteine was set as a fixed modification. Oxidation of methionine, and acetylation of the N‐terminus were set as variable modifications. The parameters for peptide length range, precursor charge range, precursor m/z range and fragment ion m/z range as well as other software parameters were used with their default values. The precursor FDR was set to 1%. Statistical analysis was performed by Perseus software, version 1.6.2.1 [32]. We used variable isolation windows throughout the scan range ranging from 10.5 to 50.5 m/z. Shorter isolation windows (10.5–18.5 m/z) were applied from 400 to 800 m/z and then gradually increased to 50.5 m/z until the end of the scan range. The default charge state was set to 3.

### 2.5 Immunoblot Analysis

Protein lysates were generated using RIPA lysis buffer (Thermofisher scientific) to which a protease inhibitors cocktail (Complete, EDTA free) and phosphoStop (Roche) were added. Protein quantification was performed using Pierce BCA Protein Assay Kits (Thermofisher scientific). Approximately 40‐50 μg of total protein was separated using 4%–15% Mini‐PROTEAN TGX Precast Protein Gels (Bio‐Rad). The gel was transferred onto a nitrocellulose membrane pre‐soaked in 1X Turbo transfer buffer (Biorad) using Trans‐Blot Turbo Transfer machine (Biorad). Membranes were blocked in 5% bovine serum albumin (BSA) (Sigma) in 1X Tris buffered saline supplemented with 0.1% Tween‐20 (Sigma) (TBS‐T) for 1–1.5 h at room temperature. The membrane was incubated with primary antibodies overnight at 4°C with gentle rocking, washed with TBST and incubated for an hour with secondary antibodies. For imaging, LiCor Odyssey imaging system (Licor UK, Crawley) is used to develop the membrane. Densitometric quantification of the bands was performed using LiCor image Studio. The following primary antibodies were used: rabbit ZEB1 (21544‐1‐AP, Proteintech, 1:1000), mouse β‐actin (SC‐47778, SantaCruz, 1:1000), rabbit YAP1 (13584‐1‐AP, Proteintech, 1:1000), rabbit VE‐cadherin (ab33168, Abcam, 1:1000), VE‐Cadherin (p‐Tyr 685) (44‐1144G, Invitrogen, 1:1000), VE‐Cadherin (p‐Tyr 731) (AB1956‐I‐200UL, Sigma, 1:1000). The secondary antibodies used were: (1:5000) antirabbit IRDye 800RD (926‐32 211, LiCor) and anti‐mouse IRDye 680RD (926‐68 070, LiCor).

### 2.6 Immunocytochemistry and Immunofluorescence Imaging

HDLECS were grown on 0.1% gelatine (Sigma) coated coverslips. Cells were washed gently with PBS and fixed with 4% formaldehyde in PBS (Thermofisher) for 10 min and 15 min. The fixative was removed, and the cells were gently washed twice with PBS. A permeabilizing solution of 0.3% triton in PBS was added for 5 min followed by blocking using 2% BSA in PBS (stock concentration of Thermo 10× blocker) for 1 h at room temperature. Cells were incubated overnight with the primary antibodies at 4°C. Cells were washed with PBS every 5 min for three times and incubated with secondary antibodies at room temperature for 1 h. Cells were washed three times then mounted on a glass slide in Vectashield and sealed using clear nail varnish. The primary antibodies used were rabbit ZEB1 (3396S, cell signaling, 1:50), mouse YAP1 (sc‐101 199, SantaCruz, 1:50), rabbit VE‐cadherin (ab33168, Abcam, 1:100), Phalloidin‐TRITC (P1951, Sigma‐Aldrich, 1:500) and secondaries (Alexa Fluor 555 goat anti‐rabbit (A21428, Invitrogen, 1:200) and Alexa Fluor 488 donkey anti‐mouse A‐21202, Invitrogen, 1:200). Imaging was performed using confocal Microscope (Leica Microsystems SP8 or SPE at 63× NA 1.3) and LAS X software (v.3.5.7.23225). ImageJ software [33] was used for image processing and analysis. To quantify “jagged junctions” [9], a BW image was generated and threshold set between 19 and 255. The length of the VE‐Cadherin positive discontinuous junctions as a percentage of total junctional length in confluent cells was calculated. 100 cells per condition were counted.

### 2.7 ECIS (Electric Cell‐Substrate Impedance Sensing)

Endothelial barrier integrity was measured using compatible E‐chips with ECIS Z‐Theta (8W1E PET and 8W10E PET and acquired 8000 Hz, sampling every 0.07 h data). Prior to the assay, cartridges/E‐chips were washed with 10 mM L‐Cysteine in PBS for 15 min. Wells were washed with PBS and incubated with a bovine type1 collagen containing solution (Advanced Biomatrix 5005, PureCol, 1:100) for 2 h. Wells were washed with PBS. RNAi on HDLECs was performed for 24 h and then 1.2 × 10^5^ cells (in 200 μL media per well per each condition (non‐silencing control and the siRNA treated cells)) were seeded. Data were analyzed using MS Excel to establish an approximately 30 min rolling average resistance, which was calculated and normalized to cell perimeter (obtained from VE‐cadherin staining).

### 2.8 Dextran Permeability Assay

24 h post transfection, 6 × 10^4^ cells were cultured in 200 μL media and were seeded into the inserts (Pore size 0.4 μm, surface area 0.33 cm^2^, 10 482 181, Corning) in the upper chamber of the wells. The lower chambers were filled with 500 μL media. Cells were left to reach confluence at 37°C in 5% CO_2_. The media in the upper chamber was then aspirated and 1 mg/mL of FITC Dextran (4 kDa) (Sigma) was added. Media from the lower chamber was taken at intervals: 4, 12, 24, 36, and 48 h. Mean fluorescence (ex:492 nm, em:525 nm) was measured with a fluorescent plate reader (Tecan Infinite M200 Pro).

### 2.9 Isolation of Monocytes From Human Peripheral Blood

Whole blood (approximately 50 mL) from anonymous healthy donors (With informed consent under and studies undertaken with ethical approvals granted by the University of Nottingham Research Ethics Committee: NB‐161‐1711 and compliant with the Declaration of Helsinki) were collected prior to starting the study. Blood was diluted 1:2 in phosphate‐buffered saline (PBS) and peripheral blood mononuclear cells (PBMCs) were separated using density gradient centrifugation though a Ficoll‐Plaque PLUS gradient (Sigma). Ficoll was centrifuged at 400*g* for 25 min with no brake at room temperature. After separation PBMCs were washed in PBS with 2% foetal calf serum (FCS) (ThermoFisher) and 1 mM EDTA (ThermoFisher). Monocytes were isolated via CD14^+^ positive selection microbeads (Miltenyi) through an autoMACs pro separator (Miltenyi). Prior to freezing monocyte purity (> 95%) was assessed through flow cytometry with anti‐CD14 and anti‐CD16 fluorescent antibodies.

### 2.10 Cryopreservation and Revival of Monocytes

Monocytes were cryopreserved by reconstituting in chilled freezing medium CryoSFM at 1 × 10^7^ cells/mL (Merck) and 500 μL aliquots were transferred into cryovials (Brooks). Cells were first stored in a −80°C freezer for 24 h before being moved to liquid nitrogen. Monocytes were cultured with benzonase (10 U/μL) in RPMI in 10% FCS for 2 h at 37°C in 5% CO_2_. Monocytes were counted and checked for viability (90%–100% viability was accepted). Monocytes were stained with 0.25 μM Deep Red cell tracker (C34565, Thermofisher Scientific) in serum‐free RPMI and left for 20 min at 37°C in 5% CO_2_. Monocytes were then washed and centrifuged. Cell pellet was the re‐suspended in endothelial cell growth medium MV2 (Promocell) supplied with MV2 supplements.

### 2.11 Leukocyte Transendothelial Migration Assay

48 h post transfection, 6 × 10^4^ cells from each condition (non‐silencing control and the siRNA treated cells) in 200 μL media were seeded into the inserts (Pore size 5 μm) (10 107 341, Corning) in the upper chamber of the wells. The lower chambers were filled with 500 μL media. Cells were left to reach confluence overnight at 37°C in 5% CO_2_. The cells were treated with 25 ng/mL TNF‐α (Peprotech) for 4 h prior to monocyte addition. Media in the upper chamber of the insert was aspirated and 5 × 10^4^ monocytes were added. Monocytes were allowed 30 min to transmigrate. Media from the lower chamber was taken. Mean fluorescence (ex:630 nm, em:660 nm) was measured with a fluorescent plate reader Tecan Infinite M200 Pro.

### 2.12 Statistical Tests

All quantitative data were analyzed using GraphPad Prism. Relative and cumulative readings (ECIS, Dextran) were plotted for each timepoint, and statistical comparisons between conditions were performed using an unpaired Student's *t*‐test. Prior to parametric testing, data distributions were assessed for normality using the Shapiro–Wilk test within GraphPad Prism to confirm suitability for parametric analysis. Where normality assumptions were met, unpaired two‐tailed Student's *t*‐tests were applied. Experiments were performed with either five or three independent biological replicates per condition, or as indicated, and results are presented as fold changes relative to control. Statistical significance was determined as *p* < 0.05.

## 3 Results

### 3.1 Silencing ZEB1 Results in Cytoskeletal Changes

**FIGURE 1:**
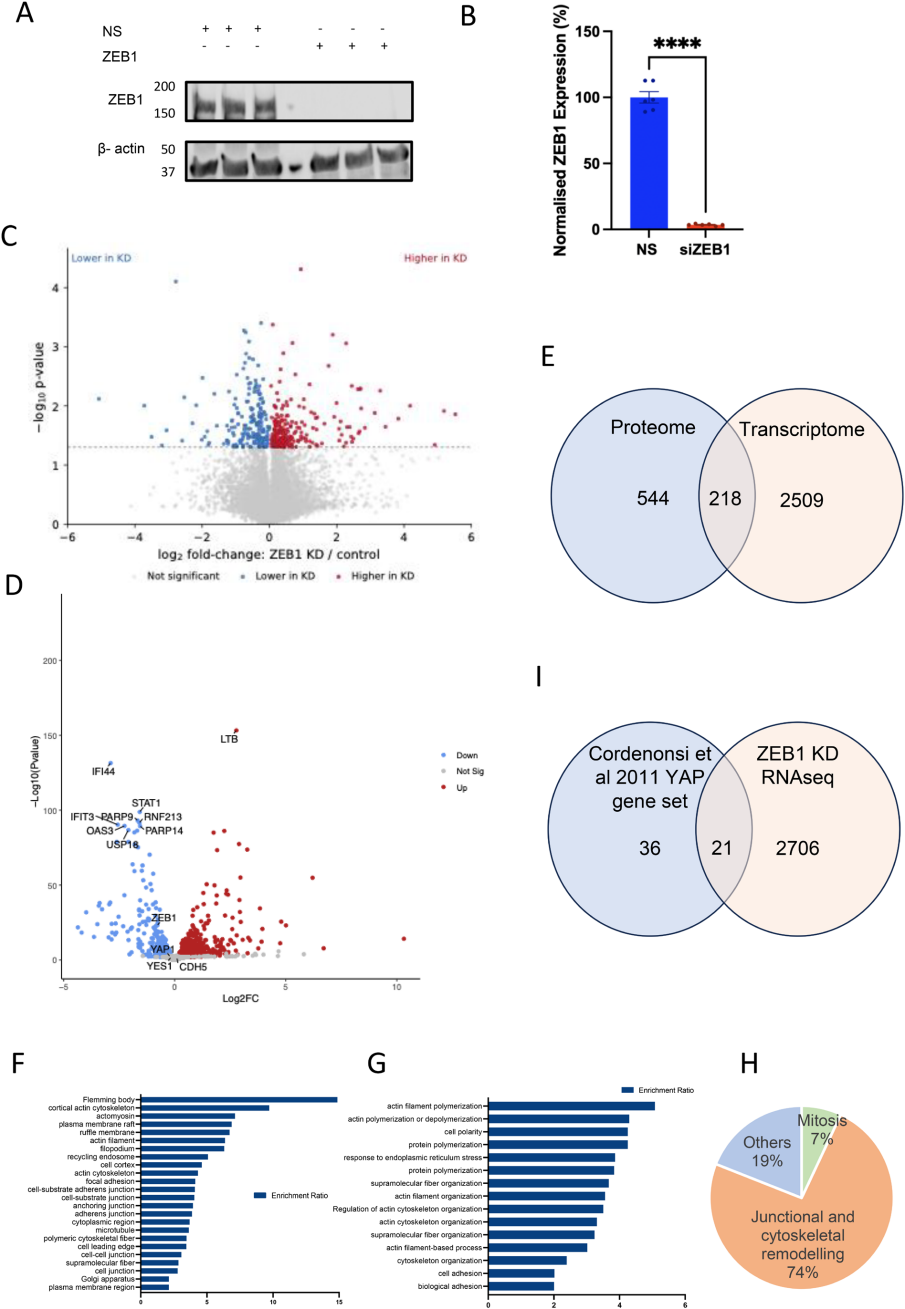
ZEB1 knockdown predicts reduced adherens junction maintenance. (A, B) HDLECs treated with siRNA to knockdown ZEB1 with densiometric quantification using Fiji ImageJ and normalization to B‐actin expression (*n* = 3). (C) Knockdown of ZEB1 shows a differential protein expression following mass spectrometric analysis. (produced on Perseus software). Colored dots indicate that DEPs that shows a significant change upon silencing ZEB1 ((−log*p*‐value > 1.3). Red dots represent upregulated DEPs while blue dots represent downregulated DEPs. Top 10 upregulated (red) and downregulated (blue) DEPs are labeled. ZEB1 lies within the top 20 downregulated DEPs. (D) Volcano plot of ZEB1KD RNAseq Control_vs_ZEB1KD with the top 10 most significant genes labeled (SPINT1 most significant) along with significant ZEB1 and YAP1, and not significant YES1 and CDH5. (E) Venn diagram showing the intersection set of differentially regulated genes/proteins in both RNA sequencing and mass spectrometric data sets. G, H) Intersected genes/proteins were interrogated to delineate GO terms based on (G) cellular compartment (H) biological process (I) Consolidation of GO Terms demonstrates the majority of altered pathways are related to adherens junctions and cytoskeletal remodeling (I) Venn diagram showing the intersection set of the differentially regulated genes in the HDLEC ZEB1KD RNAseq dataset and those in the 2011 Cordenonsi et al. paper YAP1 target gene set. Threshold was −log (*p*‐value) > 1.3. Data present as mean ± SEM. *N* = 6, analyzed using a *t*‐test, *p* < 0.0001.

As part of an unbiased investigation into zinc finger transcription factor activity in lymphatic endothelial cells (LECs), we performed siRNA‐mediated ZEB1 knockdown (KD, Figure 1A,B), followed by parallel whole‐cell proteomic and RNA‐seq analyses (Figure 1C,D, Tables S1 and S2) to identify differentially expressed proteins (DEPs) and mRNAs (DEGs), respectively. Efficient ZEB1 KD was confirmed (Figure 1A,B; *p* < 0.0001) and resulted in 762 DEPs (330 downregulated and 432 upregulated; Figure 1C) and 2728 DEGs (1323 downregulated and 1405 upregulated; Figure 1D).

Integration of both datasets (Figure S3) identified 218 common targets that were differentially regulated at both the transcript and protein levels (Figure 1E, Figure S3). Functional enrichment analysis of these overlapping targets using WebGestalt [34] revealed significant enrichment of Gene Ontology (GO) terms associated with cellular compartment (Figure 1F) and biological process (Figure 1G). Notably, 74% of the enriched GO terms related to junctional and cytoskeletal remodeling (Figure 1H). Comparison of the 2728 DEGs identified in the HDLEC ZEB1KD dataset (Figure 1E) with the YAP1 target gene set reported by Cordenonsi et al. [35] revealed overlap with 21 of the 57 genes (58%), of which 15 were downregulated and 6 were upregulated following ZEB1 KD (Figure 1H, Figure S4 with gene list).

**FIGURE 2:**
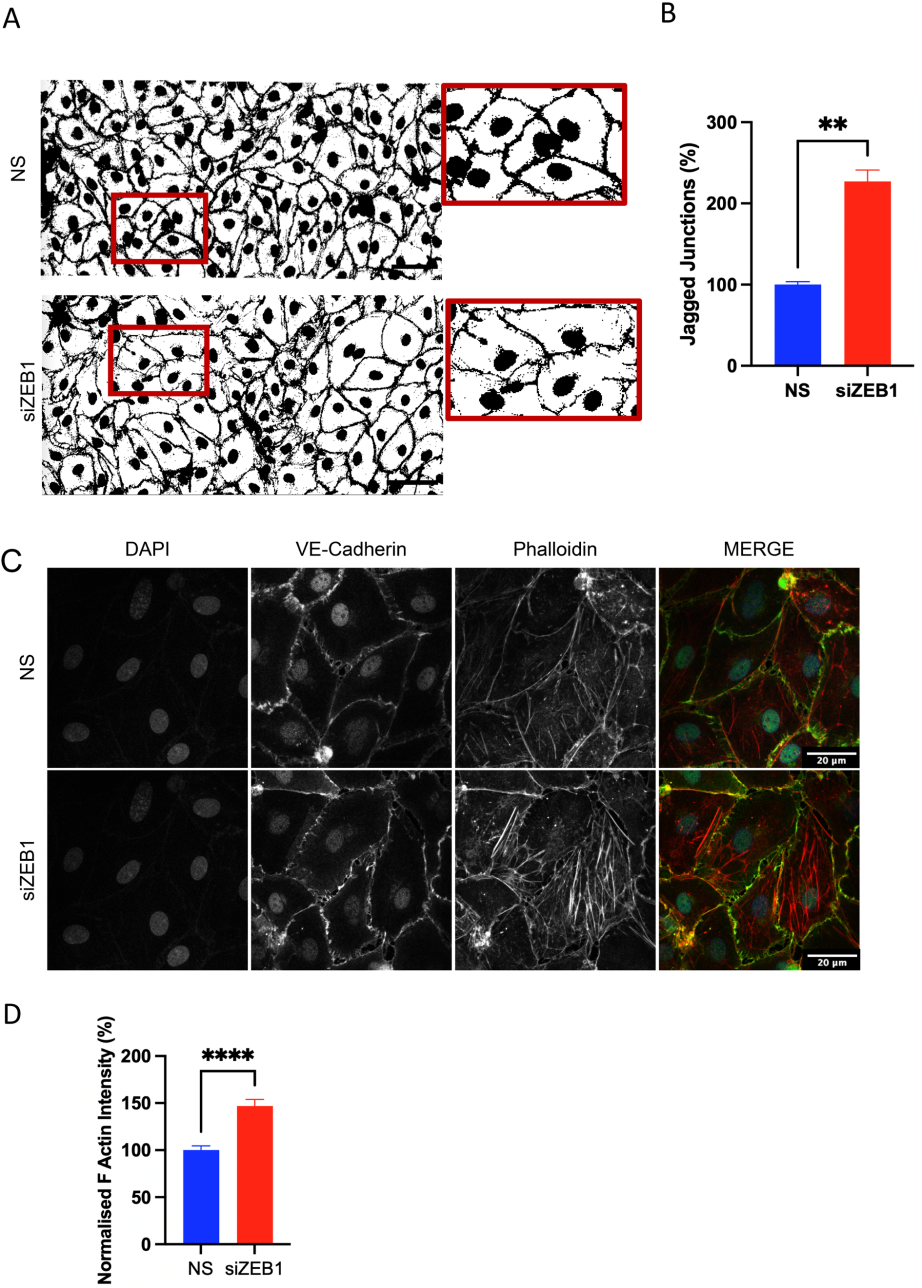
ZEB1 knockdown deficiency causes endothelium remodeling. (A–C) Immunofluorescent staining of VE = cadherin (A, B) and phalloidin (C) following ZEB1 knockdown. Number of VE‐cadherin positive “jagged junctions” (A, B) and stress fiber formation quantified (C, D). Scale bars = 20 μm (*N* = 10). Data presented as mean ± SEM, analyzed using a *t*‐test, **p < 0.01, ****p < 0.0001.

**FIGURE 3:**
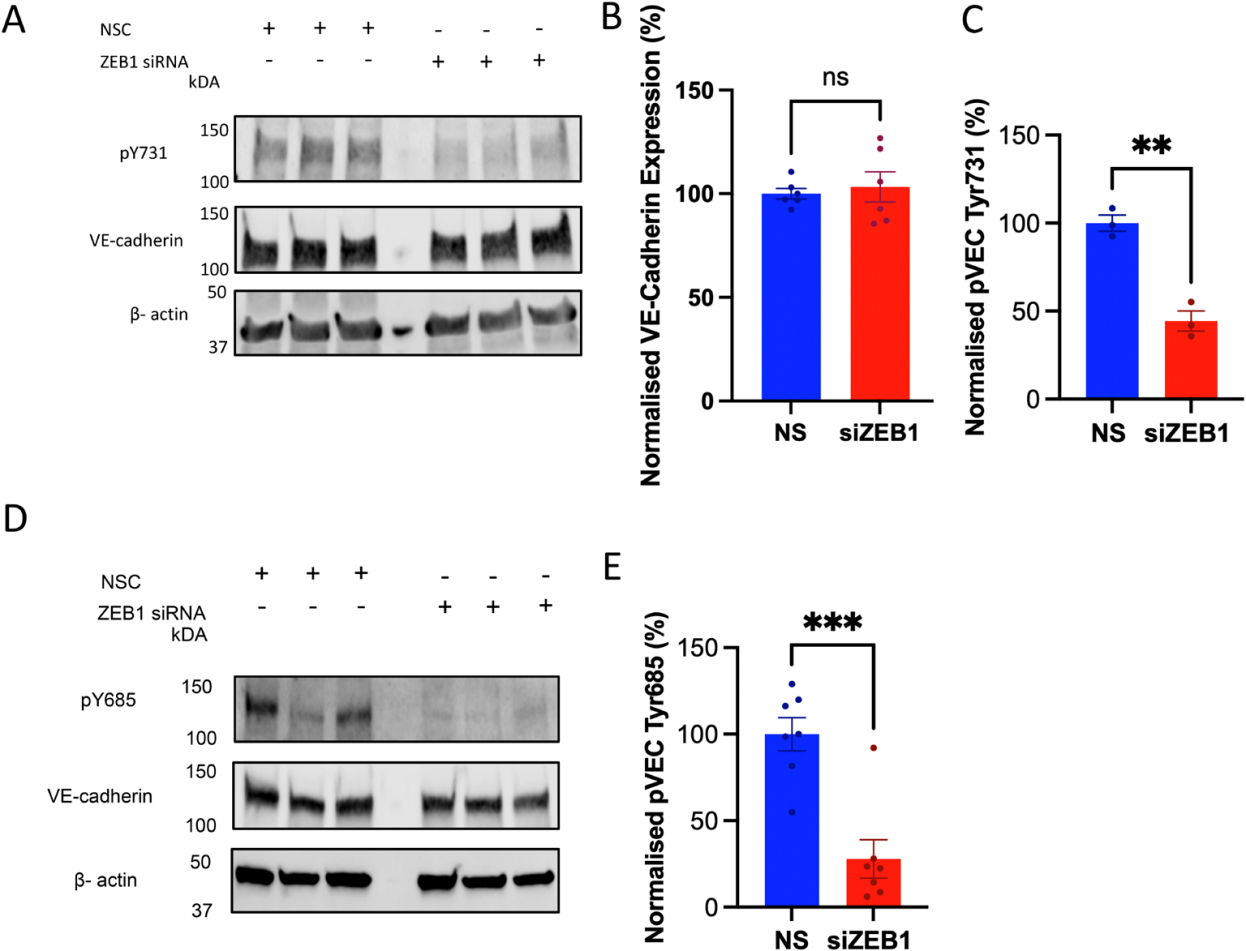
ZEB1 Knockdown reduces VE‐cadherin phosphorylation. (A–C) ZEB1 knockdown in HDLECs and western blot analysis of VE‐cadherin phosphorylation at PY731 and total VE‐cadherin normalized to VE‐cadherin and B‐actin respectively. (D, E) Densiometric quantification of VE‐cadherin phosphorylation at PY685 normalized to VE‐cadherin. Data presented as mean ± SEM. *N* = 3. Statically analyzed on GraphPad prism using unpaired *t*‐test. ***p < 0.0001; **p = 0.0008.

To experimentally corroborate these computational predictions, we examined junctional and cytoskeletal organization by immunostaining. ZEB1 KD significantly increased the proportion of “jagged junctions” relative to linear junctions (*p* = 0.0061; Figure 2A,B). This metric has recently been described in HUVECs as an indicator of disrupted VE‐cadherin signaling [9]. Consistent with altered adherens junction organization, ZEB1 knockdown also increased F‐actin staining intensity (Figure 2C,D; *p* < 0.0001). Although ZEB1 KD (Figure 1A) did not alter total VE‐cadherin protein expression (Figure 3A,B), it significantly reduced VE‐cadherin phosphorylation at Y731 (Figure 3A,C; *p* = 0.0008) and Y685 (Figure 3D,E; *p* = 0.008).

### 3.2 ZEB1 Knockdown Alters VE‐Cadherin Phosphorylation and Barrier Function

**FIGURE 4:**
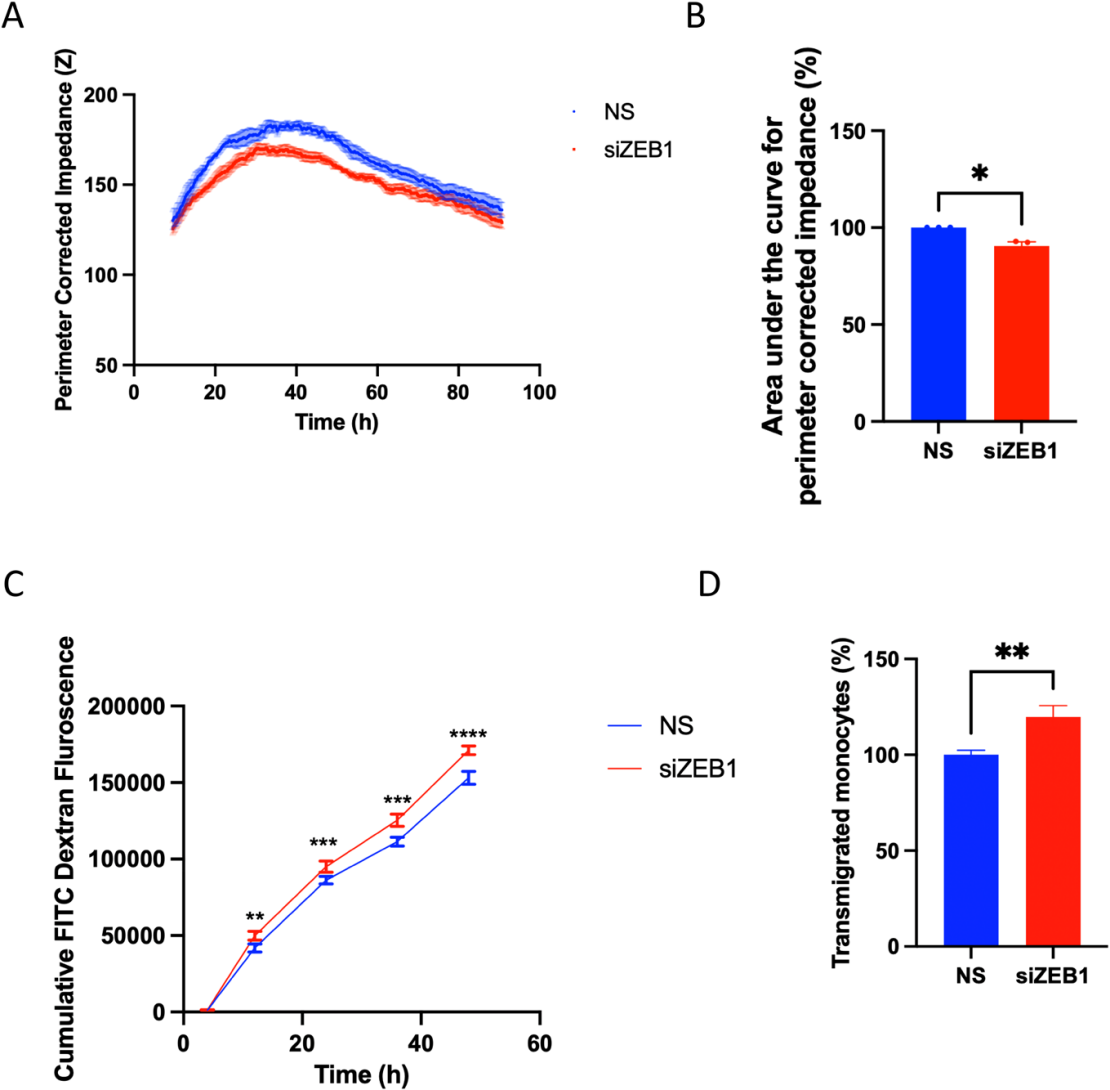
ZEB1 knockdown reduces barrier integrity. (A, B) Real time measurement of electrical impedance across a control or ZEB1 knockdown HDLEC barrier at 8 kHZ. (C, D) 4 kDa dextran leakage following ZEB1 knockdown, *p < 0.05. Furthermore, human monocyte transmigration across a HDLEC monolayer following ZEB1 knockdown monolayer. *N* = 5, data presented as mean ± SEM, analyzed by *t*‐test *p < 0.05, **p < 0.01. ***p < 0.001, ****p < 0.0001. Blue = NS, red = ZEB1 knockdown.

To assess whether ZEB1 knockdown was associated with altered endothelial barrier function, we measured LEC monolayer impedance and permeability to 4 kDa dextran. ZEB1 knockdown moderately yet significantly decreased real‐time impedance as measured by ECIS (Figure 4A,B, *p* = 0.0010) and increased 4 kDa FITC‐dextran leakage across the LEC monolayer at 12 (*p* = 0.04), 24, 36, and 48 h (*p* = 0.008) (Figure 4C). Given that Y731 phosphorylation has been reported to regulate leukocyte trafficking [10], we next assessed leukocyte transmigration. Short‐term co‐incubation of CD14^+^ monocytes (30 min) with ZEB1 knockdown LECs increased CD14^+^ transmigration (Figure 4D, *p* = 0.0087). YES tyrosine kinase has been reported to regulate VE‐cadherin phosphorylation at Y731, Y685, and Y658. We therefore examined whether ZEB1 knockdown altered YES expression.

### 3.3 Silencing ZEB1 Downregulates YAP1 Expression, Particularly Junctional YAP1

**FIGURE 5:**
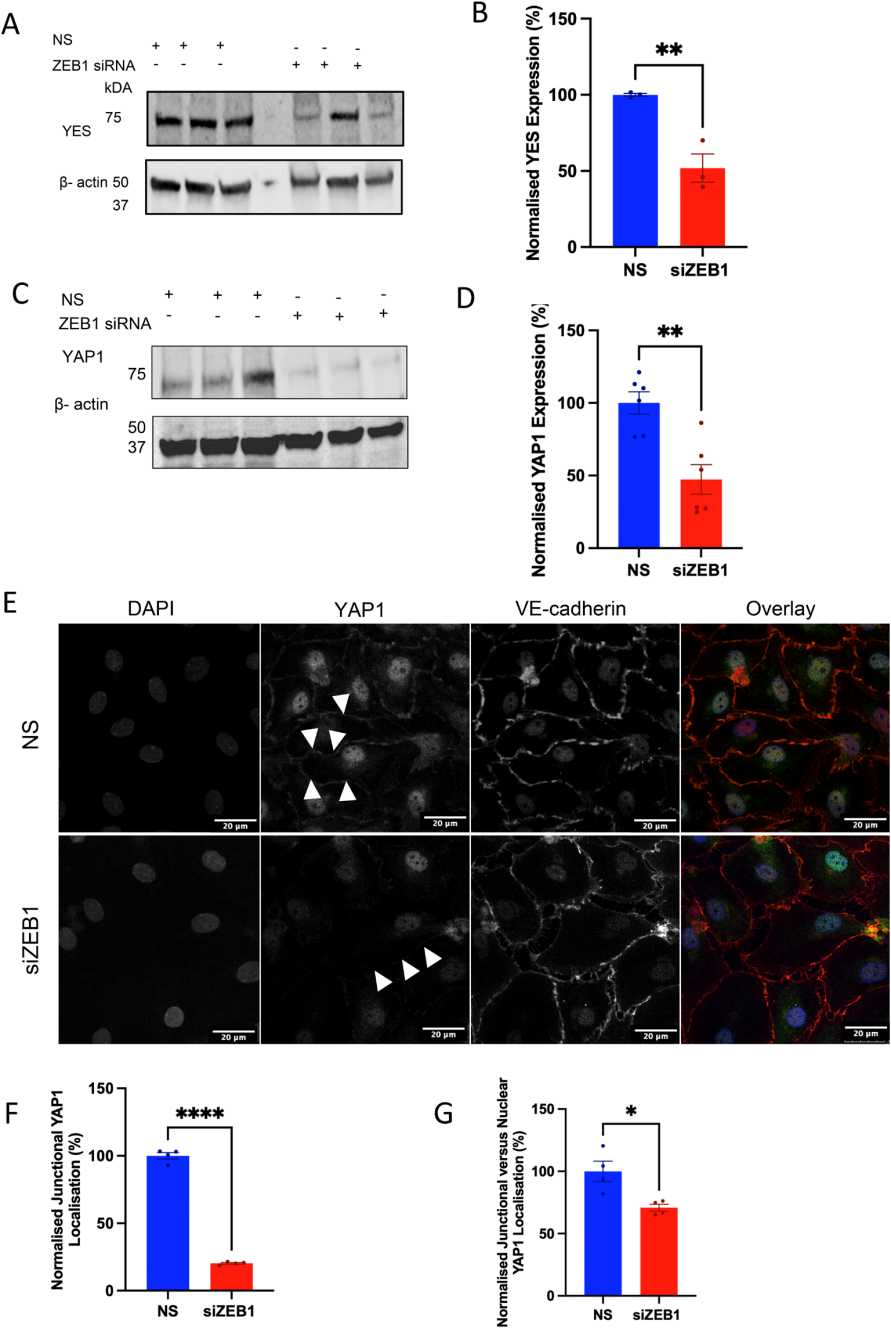
ZEB1 Knockdown reduces YES and YAP1 expression. (A–D) ZEB1 knockdown in HDLECs and western blot analysis of YES (A, B) and YAP (C, D) expression normalized to B‐actin. (E) Immunofluorescent staining of YAP1 and VE‐cadherin in ZEB1 knockdown HDLECs. (F) Quantification of YAP1 expression within inter‐EC junctions. (G) Quantification of the normalized Junctional YAP1 as a ratio of Nuclear YAP1. *p < 0.05, ****p < 0.0001, *N* = 5, analyzed by *t*‐test. (B) ** indicates *p* = 0.0068. (D) ** indicates *p* < 0.0001.

In addition to regulating VE‐cadherin phosphorylation, YES signaling also regulates the YAP/TAZ pathway [36]. ZEB1 knockdown reduced YES expression in HDLEC (Figure 5A,B, *p* < 0.05). Commonly, YAP1 acts as a nuclear acting transcriptional coactivator, however YAP1 localization has been reported in the EC junctions (Junctional YAP1 colocalizing with VE‐cadherin [37]), we therefore examined whether ZEB1 knockdown influenced YAP1 expression and localisation. ZEB1 KD reduced YAP1 expression at both the protein (Figure 5C,D, *p* < 0.0001, *t*‐test) and RNA level (DESeq2 Normalized counts, PADJ = 0.026, Figure S2). Consistent with other reports for YAP1 [9, 38, 39, 40], there was strong YAP1 localisation in the nucleus, but also at the cell–cell junctions in vitro (Figure 5E arrowheads, Figure 5F). This was further confirmed by assessing the YAP1 colocalization to junctional VE‐cadherin (Mander's coefficient, 0.579). However, upon loss of ZEB1 there was a loss of junctional YAP1 (Figure 5F,G) (80% reduction with the VE‐cadherin^+^ masked area, *p* = 0.0002).

### 3.4 YAP1 Knockdown Alters VE‐Cadherin Phosphorylation and Barrier Integrity

**FIGURE 6:**
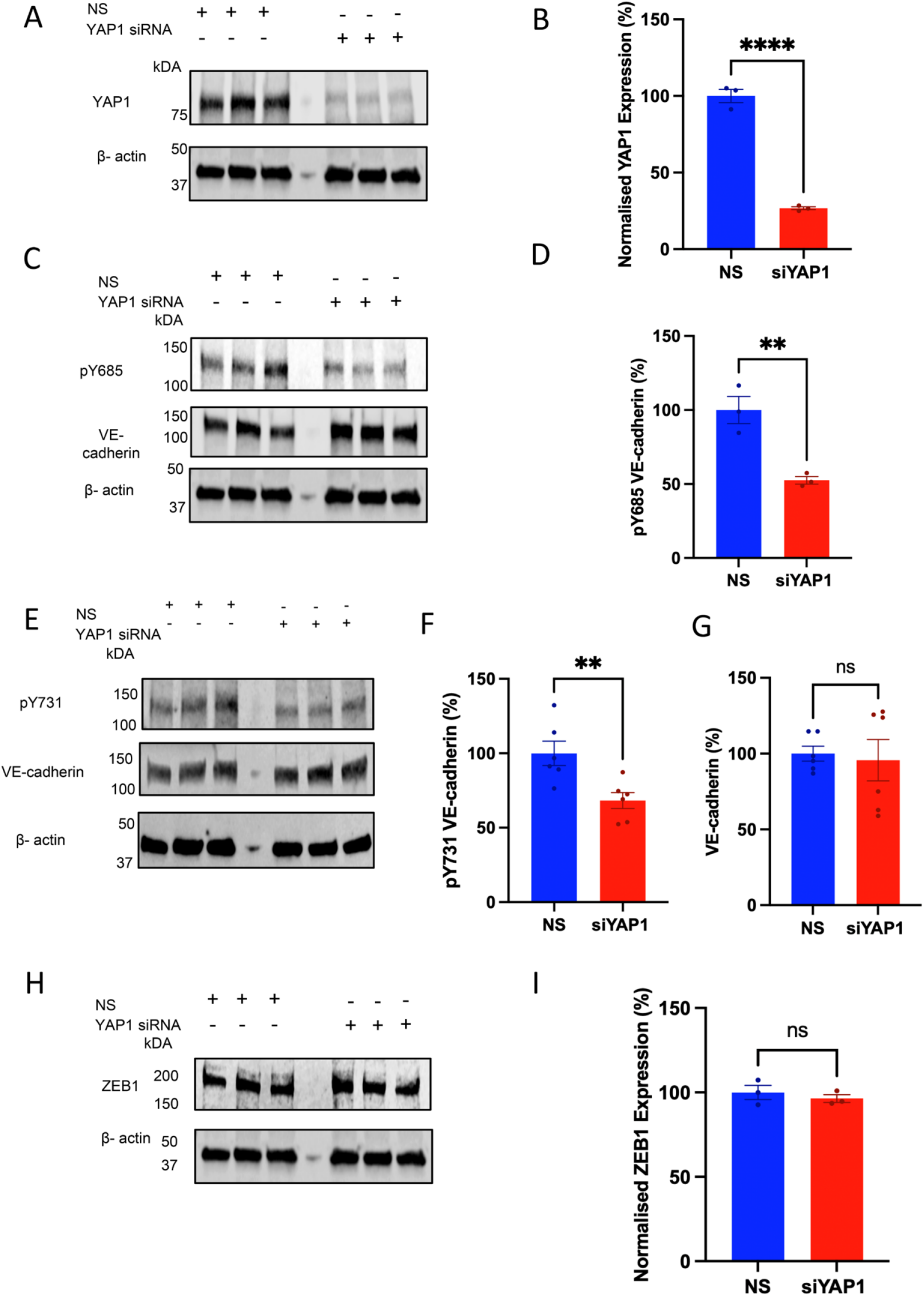
YAP1 knockdown increased junctional remodeling. (A, B) HDLECs were treated with siRNA to silence YAP1 and compared against non‐silencing control. Western blot analysis of knockdown VE‐cadherin phosphorylation at Y685 (A, C), Y731 (D, E) both normalized to VE‐cadherin, and total VE‐cadherin expression normalized to B‐actin (D, F) and ZEB1 (G, H) following YAP1 knockdown. Data presented as mean ± SEM, *N* = 6. Unpaired *t*‐test, **p < 0.01. ****p < 0.001.

**FIGURE 7:**
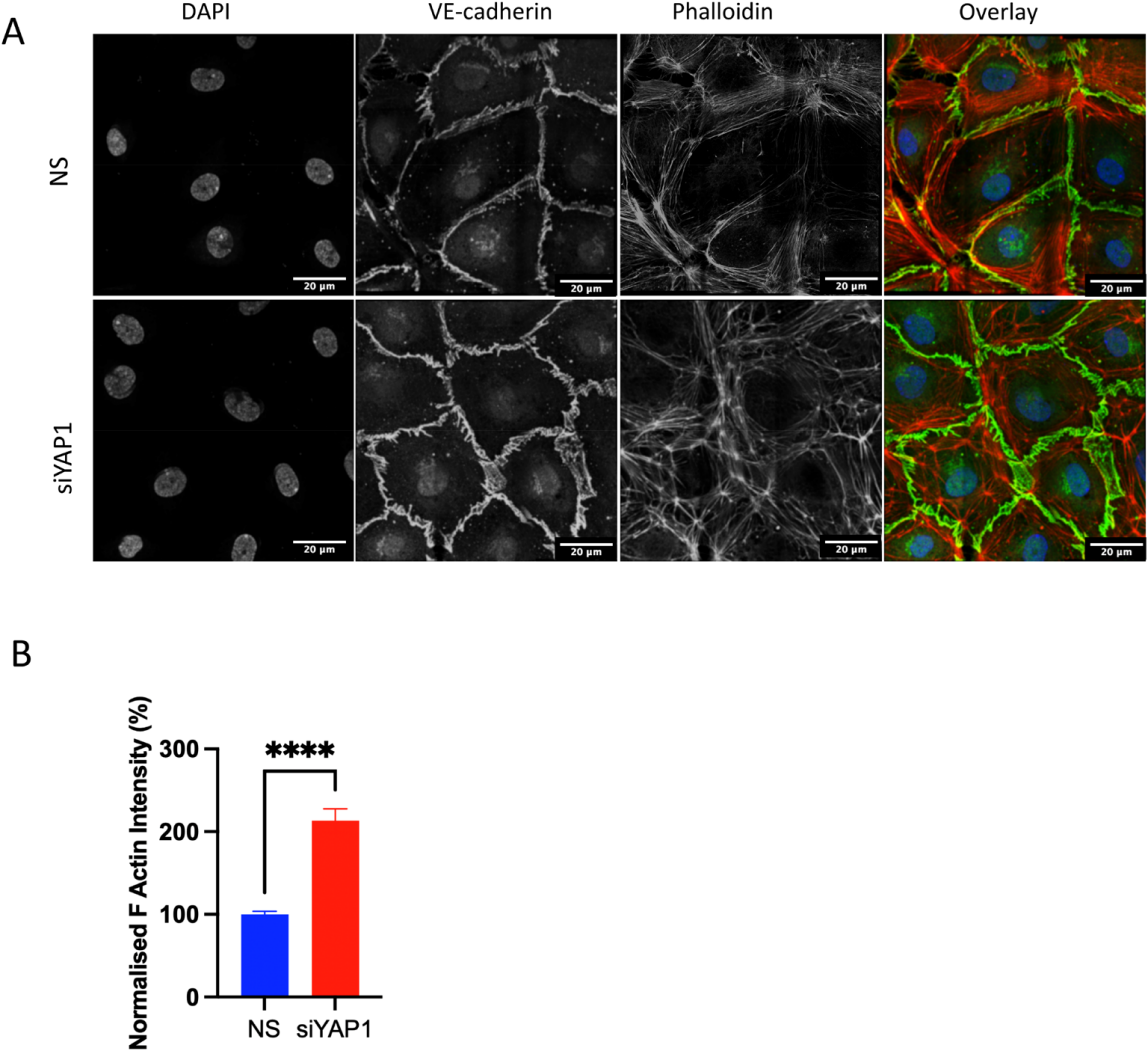
In HDLECs, silencing YAP1 affects the F‐Actin remodeling. (A) Cells stained for VE‐cadherin and F‐actin, showing the arrangement of Actin stress fibers. Scale bars, 20 μm (*N* = 12). (B) Images were acquired by Leica Microsystems SPE (63×). ImageJ is then used for image processing. 100 μm. Using GraphPad prism, results were statistically analyzed using unpaired *t*‐test, ****p < 0.001, significant.

**FIGURE 8:**
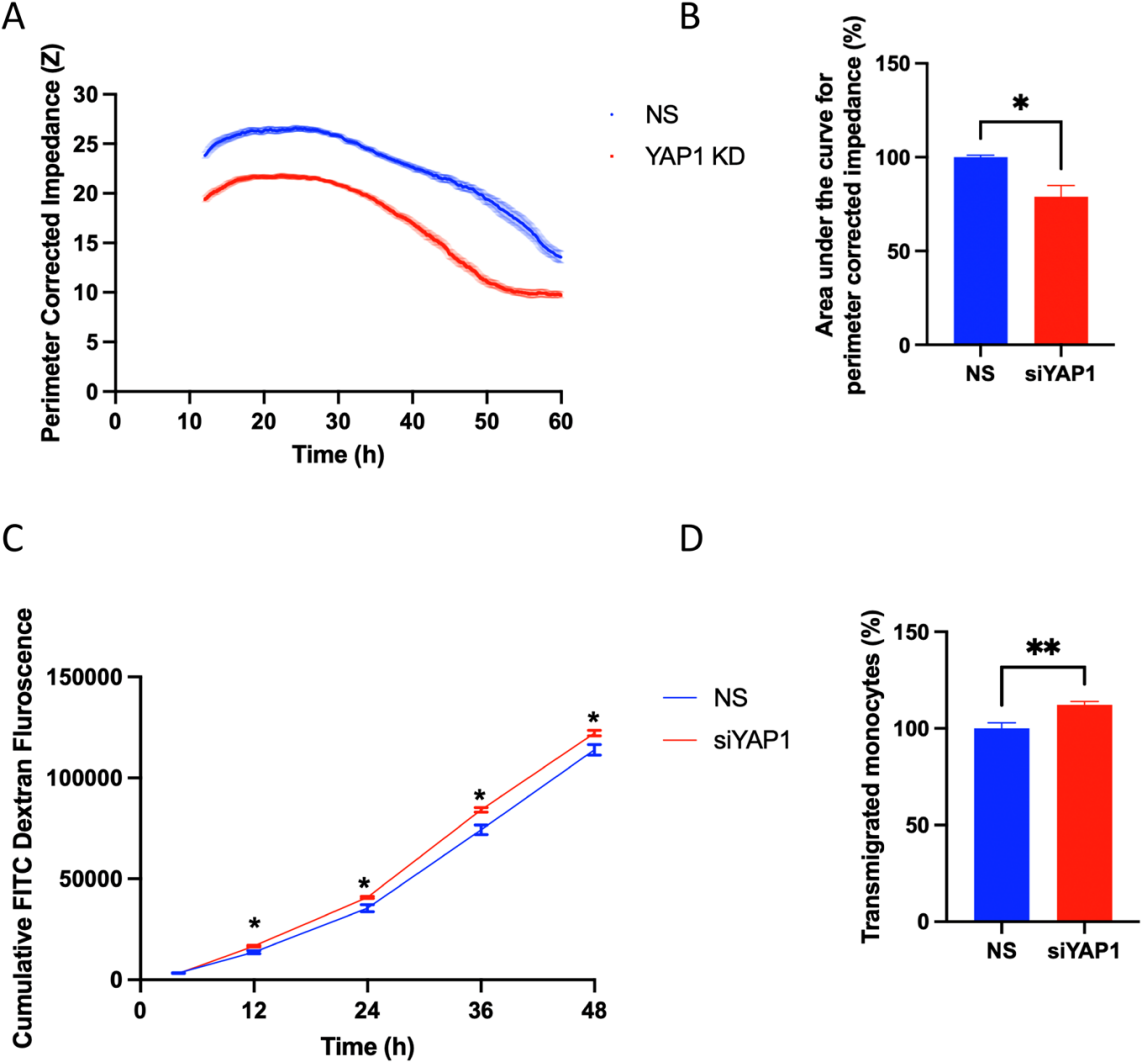
Silencing YAP1 destabilizes the junctional barrier. (A, B) Real time measurement of electrical impedance across endothelium at frequency 8 kHZ following YAP1 knockdown. (C) Endothelial leakage of 4 kDa Dextran across a HDLEC monolayer and (D) Transmigration of human monocytes through TNF‐α‐stimulated HDLECs for 30 min. *p < 0.05. **p < 0.01. Blue = NS, red = ZEB1 knockdown.

YAP1 knockdown (Figure 6A,B) did not alter total VE‐cadherin expression (Figure 6A,F, *p* > 0.05). However, stripping and reprobing of the membrane revealed significantly reduced phosphorylation at Y685 (Figure 6C,D, *p* = 0.0106) and Y731 (Figure 6E,F, *p* = 0.0084). YAP1 knockdown did not alter ZEB1 expression (Figure 6H,I), consistent with ZEB1 acting upstream of YAP1. In parallel, YAP1 knockdown increased F‐actin‐positive stress fiber formation (Figure 7A,B, *p* < 0.0001), reduced monolayer resistance (Figure 8A,B, *p* = 0.01), increased 4 kDa FITC‐dextran leakage at 12 (*p* = 0.0025), 24 (*p* = 0.0231) and 36 h (*p* = 0.0026) (Figure 8C), and enhanced transendothelial migration (Figure 8D, *p* = 0.0023). Collectively, these findings are consistent with ZEB1 contributing to barrier maintenance in LECs in vitro in association with altered YAP1 and YES signaling and reduced VE‐cadherin phosphorylation.

## 4 Discussion

Endothelial barrier integrity is governed by the dynamic regulation of VE‐cadherin activity, including its assembly with binding partners and coregulators, and its phosphorylation status, all of which can be spatially and temporally modified [11, 37, 41, 42]. In this study we examined the consequences of reduced ZEB1 expression in regulating: (1) inter‐LEC junction morphology, (2) VE‐cadherin phosphorylation status, (3) junctional integrity, and (4) YAP1/YES signaling cross talk, a known modifier of VE‐cadherin signaling.

ZEB1 signaling has recently been described as playing several roles in angiogenic endothelial cells [16, 18, 43], but its function in lymphatic endothelial cells has not previously been examined. ZEB1 has been reported to negatively regulate tumor angiogenesis, and ZEB1 knockdown in HUVECs reduces tube stability and increases vessel fragility [15]. Furthermore, endothelial ZEB1 knockout promotes vessel quiescence during later stages of neonatal retinal remodeling [16] but conversely promotes tumor angiogenesis [15]. Our findings suggest an additional mechanism through which ZEB1 may regulate endothelial quiescence. Specifically, we show that ZEB1 contributes to the maintenance of VE‐cadherin‐mediated junctional integrity, a process that appears to involve regulation of YES kinase signaling.

Consistent with this, enriched DEGs and DEPs identified following ZEB1 knockdown in HDLECs were linked to processes regulating endothelial barrier stability (Figure 1). ZEB1 KD reduced VE‐cadherin phosphorylation at Y685 and Y731, changes that are associated with reduced barrier resistance and increased vascular leakage, thereby facilitating monocyte transendothelial migration. Wessel et al. demonstrated that VE‐cadherin phosphorylation is critically linked to endothelial junction integrity [10]. VE‐cadherin is the canonical marker of inter‐EC junctions [44], and its phosphorylation regulates nuclear accumulation of unphosphorylated FOXO1 and β‐catenin to control claudin‐5 localisation and cell–cell adhesion [45].

Although dephosphorylation of Y731 is widely associated with reduced monocyte extravasation, Wessel et al. also highlighted the importance of leukocyte exposure duration [10, 46]. Short term leukocyte exposure (approximately 30 min) rapidly induces Y731 dephosphorylation and promotes transendothelial migration. Consistent with this model, short term co incubation of CD14+ monocytes with siZEB1 HDLECs significantly increased transendothelial migration and was associated with Y731 dephosphorylation. In addition, Jin et al. demonstrated that loss of VE cadherin phosphorylation at Y685 promotes vascular leakage through increased permeability sites in endothelial specific YES knockout mouse rretinas [17]. Consistent with these findings, we observed increased endothelial leakage following ZEB1 knockdown, accompanied by reduced VE cadherin phosphorylation and decreased YES expression. Although reduced VE‐cadherin phosphorylation in our study was associated with increased permeability, earlier reports, particularly in blood microvascular endothelial cells, have described increased tyrosine phosphorylation of VE‐cadherin [47] in response to acute VEGF stimulation as promoting vascular leakage. This apparent discrepancy likely reflects context‐ and site‐specific differences in VE‐cadherin regulation [48]. Tyrosine phosphorylation of VE‐cadherin is not a uniform event but occurs at multiple residues, including Y658, Y685 and Y731, each of which can exert distinct effects on junctional stability, leukocyte transmigration and endothelial permeability. In addition, many early studies examined acute growth factor–induced permeability in blood endothelial cells, whereas our model involves sustained transcription factor depletion in primary lymphatic endothelial cells under basal conditions. Lymphatic endothelial junctions also differ structurally and functionally from those in the blood vasculature, exhibiting specialized button and zipper configurations that may respond differently to changes in phosphorylation dynamics [44]. Together, these considerations suggest that VE‐cadherin phosphorylation should be interpreted within the specific cellular and temporal context [8], and that both increases and decreases in phosphorylation at defined residues may disrupt normal junctional homeostasis. Although YES has been reported to phosphorylate VE‐cadherin in other endothelial contexts, we did not directly assess kinase activity in HDLECs, and therefore cannot exclude contributions from additional kinases. Together, these results suggest that YES may act downstream of ZEB1 and contribute to the maintenance of endothelial junctional integrity.

YAP1 is a downstream effector of the Hippo pathway in which the active, dephosphorylated form of YAP1 translocates to the nucleus and interacts with TEAD transcription factors to regulate gene programmes controlling differentiation, migration, and proliferation [49, 50, 51]. In addition to its transcriptional roles, YAP1 has recently been shown to localize at endothelial junctions where it regulates vessel permeability and angiogenic behavior [17, 52, 53]. Importantly, YAP1 has also been identified as a determinant of YES kinase activity, as demonstrated by the profound repression of proliferation in YES Y537F mutant mice following YAP1 depletion. These observations suggest that regulation of YAP1 expression and localisation is critical for maintaining vascular integrity [36, 39].

The overlap between the downregulated genes identified in our ZEB1 KD RNAseq dataset and the YAP1 target gene set described by Cordenonsi et al. suggests that ZEB1 may influence kinase signaling pathways through YAP1 dependent mechanisms [35]. YAP1 localisation is regulated by nuclear cytoplasmic shuttling and by phosphorylation through Hippo pathway kinases such as LATS1 and LATS2, which promote cytoplasmic retention and inhibit transcriptional activity [54, 55]. YAP1 signaling is also sensitive to cytoskeletal organization, mechanical forces, and Rho GTPase driven actin dynamics [37, 56, 57]. Genetic studies further support an essential vascular role for YAP1 signaling: YAP1 TAZ knockout causes embryonic lethality in zebrafish due to severe vascular defects, while endothelial YAP1 deletion in mice leads to blood vessel rupture, regression, and heart valve abnormalities [58]_′_ [59, 60]. During angiogenesis in the mouse retina, YAP1 expression is enriched at the angiogenic front and becomes more diffuse in the vascular plexus as remodeling progresses. Suppression of YAP1 during vascular remodeling reduces vascular branching and density through repression of Angiopoietin‐2 [61].

In our integrated analysis, 218 targets were significantly altered at both the transcript and protein level following ZEB1 knockdown, representing approximately 29% of differentially expressed proteins and 8% of differentially expressed transcripts. Although this overlap may appear modest, such proportions are consistent with established RNA protein concordance rates in mammalian systems, which typically range between approximately 20% and 40% [62] These differences arise from post transcriptional regulation, variation in translational efficiency, protein stability and turnover, and temporal delays between transcriptional and proteomic responses. Transcriptomic and proteomic datasets should therefore be considered complementary layers of biological regulation rather than direct validation platforms. Indeed, most proteomic alterations occurred independently of detectable transcript changes, suggesting that ZEB1 dependent regulation in LECs extends beyond direct transcriptional control and likely involves secondary signaling cascades and protein network modulation. The behavior of YAP1 and YES illustrates this principle. While YAP1 reached statistical significance at the transcript level and both YAP1 and YES proteins were reproducibly reduced by targeted immunoblotting, neither reached significance thresholds in the unbiased proteomic analysis. Mass spectrometry based proteomics is inherently biased toward higher abundance proteins and involves stringent multiple testing correction across thousands of analytes, meaning that regulatory signaling proteins may fall below detection thresholds. Targeted validation therefore provides greater sensitivity for individual signaling proteins and supports the biological relevance of these ZEB1 dependent changes.

The mechanisms by which ZEB1 regulates YAP1 remain unclear and are beyond the scope of this study. However, we observed a modest reduction in YAP1 transcript levels following ZEB1 knockdown (fold change = 0.84) accompanied by a more pronounced reduction in YAP1 protein expression. YAP1 expression is known to be influenced by cell–cell contact [40], with higher cell density promoting its expression, and by altered flow conditions, cell density, and flow‐mediated signaling, and its junctional localisation contributes to flow‐dependent regulation of endothelial permeability [58]. Consistent with these roles, YAP1 silencing in our system disrupted junctional integrity and increased endothelial permeability and leukocyte transendothelial migration in vitro, recapitulating the phenotype observed following ZEB1 depletion. Importantly, YAP1 suppression produced a VE‐cadherin phosphorylation pattern similar to that observed following disruption of ZEB1 YES signaling, suggesting that YES kinase activity may be partially dependent on YAP1. Specifically, YAP1 deficiency significantly reduced VE‐cadherin phosphorylation at Y685 and, to a lesser extent, at Y731.

Although our experiments were performed in vitro using primary human dermal lymphatic endothelial cells, it is important to consider how these findings may relate to lymphatic barrier function in vivo. HDLECs cultured under static, confluent conditions most closely resemble collecting or zipper‐like lymphatic endothelium, rather than the specialized button junctions of initial lymphatic capillaries, which facilitate interstitial fluid uptake through discontinuous junctional organization [63]. In contrast, collecting lymphatic vessels form continuous zipper junctions that maintain controlled barrier integrity during lymph transport [44, 63].

Regulation of lymphatic permeability has been less extensively characterized than in blood vessels, but emerging evidence indicates that VE‐cadherin phosphorylation, cytoskeletal tension and inflammatory signaling influence lymphatic junction stability in a context‐dependent manner [8, 42, 44]. Inflammatory cytokines and mechanical forces have been shown to modulate lymphatic endothelial barrier properties, suggesting that phosphorylation‐dependent remodeling of adherens junctions may contribute to adaptive changes in permeability [8, 40].

Although lymphatic vessels are classically associated with leukocyte entry from tissues into the lymphatic lumen, dynamic transmigration across lymphatic endothelium has been well described, particularly under inflammatory conditions [40, 42, 64]. Dendritic cells and monocytes actively traverse lymphatic endothelial junctions during immune surveillance and resolution of inflammation [64]. Our findings therefore suggest that ZEB1‐dependent regulation of VE‐cadherin phosphorylation and junctional organization may contribute to the dynamic control of lymphatic barrier properties, particularly within collecting lymphatics or inflamed lymphatic beds. However, in vivo validation will be required to determine the specific lymphatic compartments and physiological contexts in which ZEB1‐dependent signaling exerts functional relevance.

## 5 Perspectives

Collectively, these findings suggest that the interplay between YES kinase and YAP1 represents an important component of ZEB1‐mediated regulation of VE‐cadherin phosphorylation and junctional stability in lymphatic endothelial cells.

## Author Contributions

Nada S. Ahmed, Joseph L. Horder, Amy P. Lynch, Zarah B. Tabrizi, Charles T. Cresswell, Poppy E. Harris, Kathryn R. Green, James H. Hallwood, Michael A. Portelli, Alexander J. Fezovich, Christos Spanos, Sarah J. Storr, Andrew V. Benest performed experiments and analysis. Nada S. Ahmed, David S. Gardner, Alan McIntyre, Sarah J. Storr, Daniel G. Booth, David O. Bates, and Andrew V. Benest designed experiments and drafted the manuscript.

## Funding

This work was supported by British Heart Foundation. Wellcome Trust, Royal Society.

## Conflicts of Interest

The authors declare no conflicts of interest.

## Supporting information


**Data S1:** Whole Cell Proteomics data, ZEB1 knockdown results compared with NS control samples.

**Data S2:** List of PADJ sorted genes following RNAseq analysis of ZEB1 KD compared with NS control samples.

**Data S3:** Intersected genes, identified as being significantly changed following ZEB1 knockdown in both proteomic and RNA sequencing techniques.

**Data S4:** overlapping DEG of YAP and ZEB1 signaling.

## Data Availability

The data that support the findings of this study will be openly available in GEO and ProteomeXchange, reference number [Awaitng reference number]. All other data are available from the corresponding author upon reasonable request. Specifically, the RNAseq data generated in this study have been deposited in the Gene Expression Omnibus (GEO) repository under accession number [awaiting accession number], available at [https://www.ncbi.nlm.nih.gov/geo/]. The proteomic data have been deposited in the ProteomeXchange Consortium via the Proteomics Identification Database (PRIDE) with the dataset identifier [awaiting dataset identifier], available at https://www.proteomexchange.org. These data are openly available and can be accessed without any restrictions. We encourage researchers to utilize and build upon these datasets for further exploration and advancement in the field.
